# Prognostic value of baseline imaging and clinical features in patients with advanced hepatocellular carcinoma

**DOI:** 10.1038/s41416-021-01577-6

**Published:** 2021-10-22

**Authors:** Osman Öcal, Michael Ingrisch, Muzaffer Reha Ümütlü, Bora Peynircioglu, Christian Loewe, Otto van Delden, Vincent Vandecaveye, Bernhard Gebauer, Christoph J. Zech, Christian Sengel, Irene Bargellini, Roberto Iezzi, Alberto Benito, Maciej Pech, Peter Malfertheiner, Jens Ricke, Max Seidensticker

**Affiliations:** 1 Department of Radiology, University Hospital, LMU Munich, Munich, Germany; 2 Department of Radiology, Hacettepe University, Ankara, Turkey; 3 Section of Cardiovascular and Interventional Radiology, Department of Bioimaging and Image-Guided Therapy, Medical University of Vienna, Vienna, Austria; 4 Department of Radiology and Nuclear Medicine, Academic Medical Center, University of Amsterdam, Amsterdam, The Netherlands; 5 Department of Radiology, University Hospitals Leuven, Leuven, Belgium; 6 Department of Radiology, Charité—University Medicine Berlin, Berlin, Germany; 7 Radiology and Nuclear Medicine, University Hospital Basel, University of Basel, Basel, Switzerland; 8 Radiology Department, Grenoble University Hospital, La Tronche, France; 9 Department of Vascular and Interventional Radiology, University Hospital of Pisa, Pisa, Italy; 10 Fondazione Policlinico Universitario A. Gemelli IRCCS, UOC di Radiologia, Dipartimento di Diagnostica per Immagini, Radioterapia Oncologica ed Ematologia, Roma, Italia; 11 Abdominal Radiology Unit, Department of Radiology, Clínica Universidad de Navarra, Pamplona, Spain; 12 Departments of Radiology and Nuclear Medicine, University of Magdeburg, Magdeburg, Germany; 13 Department of Medicine II, University Hospital, LMU Munich, Munich, Germany

**Keywords:** Hepatocellular carcinoma, Prognostic markers

## Abstract

**Aims:**

To investigate the prognostic value of baseline imaging features for overall survival (OS) and liver decompensation (LD) in patients with hepatocellular carcinoma (HCC).

**Design:**

Patients with advanced HCC from the SORAMIC trial were evaluated in this post hoc analysis. Several radiological imaging features were collected from baseline computed tomography (CT) and magnetic resonance imaging (MRI) imaging, besides clinical values. The prognostic value of these features for OS and LD (grade 2 bilirubin increase) was quantified with univariate Cox proportional hazard models and multivariate Least Absolute Shrinkage and Selection Operator (LASSO) regression.

**Results:**

Three hundred and seventy-six patients were included in this study. The treatment arm was not correlated with OS. LASSO showed satellite lesions, atypical HCC, peritumoral arterial enhancement, larger tumour size, higher albumin–bilirubin (ALBI) score, liver–spleen ratio <1.5, ascites, pleural effusion and higher bilirubin values were predictors of worse OS, and higher relative liver enhancement, smooth margin and capsule were associated with better OS. LASSO analysis for LD showed satellite lesions, peritumoral hypointensity in hepatobiliary phase, high ALBI score, higher bilirubin values and ascites were predictors of LD, while randomisation to sorafenib arm was associated with lower LD.

**Conclusions:**

Imaging features showing aggressive tumour biology and poor liver function, in addition to clinical parameters, can serve as imaging biomarkers for OS and LD in patients receiving sorafenib and selective internal radiation therapy for HCC.

## Introduction

Hepatocellular carcinoma (HCC) comprises 75–85% of all liver cancers and is the fourth most common cause of cancer-related death in the world [1]. Patients amenable to curative treatments such as surgical resection, liver transplantation or percutaneous ablation have 5-year survival rates of 50–75%, but, despite screening high-risk patients, most of the cases are present at intermediate or advanced stages [2].

Sorafenib has been shown to improve the overall survival (OS) of HCC patients compared to placebo in two randomised trials, and since then, it has been the standard treatment for advanced HCC cases [3, 4]. Although trials comparing selective internal radiation therapy (SIRT) with sorafenib in intermediate-advanced HCC cases failed to show a survival benefit, they demonstrated higher tolerability of SIRT and the ability of SIRT to serve as an effective alternative therapy in patients who are not able to receive sorafenib [5, 6]. Also, in the SORAMIC trial, the addition of SIRT to sorafenib did not improve OS compared to sorafenib alone. However, subgroup analysis has shown increased benefit for some patient groups, including younger age, non-cirrhotic liver or non-alcoholic aetiology [7]. These results are compatible with the previous studies showing significant heterogeneity in outcomes of patients treated with sorafenib or SIRT [8].

Several clinical factors for improved outcomes have been investigated, and patients with low tumour burden and preserved liver function have been shown to benefit more than other patients [9]. However, except for the size and obvious macrovascular invasion of the lesion, none of the imaging appearances has been utilised in available clinical staging systems (i.e. Barcelona Clinic Liver Cancer [BCLC] classification or Child–Pugh scores). Patients with the same BCLC class or Child–Pugh grade do not benefit from the treatments equally, and there is a need for additional features to cover all the variations in clinical outcomes within the same category. Multiple studies have investigated a correlation between radiological appearances and recurrence, survival or histopathological grade of the lesions [10, 11], but most of these studies investigated patients who underwent resection or transplantation. Evaluation of such imaging features after SIRT or sorafenib treatment is lacking in the literature.

Some predictors of biological behaviour have been identified based on the evaluation of resected HCC lesions. For example, microvascular invasion (mVI) has been shown to correlate with early recurrence after surgery [12]. However, in lesions with typical enhancement characteristics, histopathological evaluation is not mandatory, and most of the patients with advanced HCC do not undergo a biopsy before treatment. Even if a histopathological evaluation is done, especially in patients with a heterogeneous lesion, biopsy does not represent the whole tumour and may miss the liver interface with a possible display of mVI. However, several reports have shown that some imaging features are correlated with mVI and further descriptors of biological behaviour of the tumour (i.e. satellite lesions) as well as liver function status (e.g. presence of ascites or hepatobiliary phase enhancement) [11, 13].

This post hoc analysis of the SORAMIC trial was conducted to explore the correlation of baseline radiological imaging features related to the tumour as well as liver function with OS and time to deterioration in liver function.

## Materials and methods

### Study population

The present study was performed within the palliative part of SORAMIC trial (EudraCT 2009-012576-27, NCT01126645), a prospective, randomised-controlled, phase II trial comparing the effects of sorafenib monotherapy and the combination of SIRT and sorafenib, conducted at 38 cites in 12 countries in Europe and Turkey. The main inclusion criteria were age between 18 and 85 years, a diagnosis of HCC with histopathology or EASL imaging criteria, preserved liver function (Child–Pugh scores A to B7) and an Eastern Cooperative Oncology Group performance status ≤2. Extrahepatic metastases were permitted as long as lungs were not involved and the disease was liver-dominant.

Patients underwent computed tomography (CT) and magnetic resonance imaging (MRI) with a standardised imaging protocol according to the diagnostic study of the SORAMIC trial [14]. In summary, the CT protocol consisted of pre-contrast (native) images followed by arterial (15 s), portovenous (50 s) and venous (delayed) phase (120 s) images with a slice thickness of ≤5 mm after intravenous injection of the contrast material. For MRI, besides dynamic series, axial T2 images and hepatobiliary phase images obtained 20 min after the injection of gadoxetic acid were mandatory. Pre-treatment CT images were available for central review in 378 of 424 patients randomised within the palliative arm of the SORAMIC trial. Two patients were excluded due to missing arterial phase images. A total of 376 patients were included in the study (Supplementary Fig. 1). For 365 of these patients, MRI images were also present. While 192 of 376 patients were randomised to the combination arm, 184 patients received sorafenib monotherapy.

### Image analysis

Radiological parameters were evaluated in pre-treatment abdomen CT and liver MRI images by a radiologist with specialisation in liver imaging for >6 years blinded to the treatment arm and all clinical information. All of the lesions were evaluated in terms of enhancement pattern, and the presence of a lesion with atypical enhancement was recorded. Typical enhancement was defined as enhancement in the arterial phase (wash-in) and lower contrast uptake than liver parenchyma (wash-out) in portal venous or venous phase images.

The largest lesion was chosen as an index lesion and further analysis was done with this lesion. The size of the lesion was measured in the venous phase on CT and hepatobiliary phase on MRI. For both imaging modalities, lesion margins and the presence of macrovascular invasion, biliary dilatation, extrahepatic disease, gastroesophageal varices, ascites, pleural effusion and satellite tumour were evaluated. A satellite tumour was defined as a lesion smaller than 2 cm with a distance <2 cm to another HCC lesion [11]. Lesion margins of simple nodular lesions with regular contours were regarded as smooth and margins of multinodular lesions or lesions with extranodular confluent growth were regarded as irregular [15]. The presence of peritumoral enhancement in the arterial phase, which becomes isodense on the venous/delayed phase, and the presence of complete peripheral ring enhancement (capsule) was evaluated in the venous/delayed phase in both modalities [15]. The fat deposition was assessed on native CT images and opposed-phase MR images. Furthermore, in CT images on the same slice as the diameter measurement, the lesion’s Hounsfield unit (HU) value was measured on every phase (native, arterial, portal and venous) with as large as possible a region of interest confined within the tumour excluding major necrotic areas. In case of artefacts preventing image analysis in a phase, measurement on that series was omitted.

### MRI-specific parameters

In the hepatobiliary phase, on the same slice as the diameter measurement, the signal intensity (SI) of the lesion and healthy liver tissue was evaluated. On the corresponding slice of native T1 images, the same measurements were repeated. The SI of the spleen was also evaluated in the same phases. Using these measurements, liver–spleen ratio (SI of healthy liver/SI of the spleen in hepatobiliary phase, LSR) and relative liver enhancement ([SI of the liver in hepatobiliary phase − SI of the liver in native images]/SI of the liver in native images, RLE) were calculated using these measurements. LSR was dichotomised according to an established cut-off value of 1.5 [16]. On hepatobiliary images, lesion intensity was also semiquantitatively graded with reference to the liver parenchyma as low, iso, high or mixed, and the presence of hypointensity adjacent to the lesion was evaluated as a descriptor of mVI [17]. For 208 patients, diffusion-weighted imaging (DWI) images were available, and lesion intensity was graded semiquantitatively as low, iso or high with reference to the liver parenchyma on the highest *b* value. Apparent diffusion coefficient (ADC) values were calculated on the slice with the largest tumour, excluding major necrosis.

In order to evaluate interreader reproducibility of all imaging parameters, 100 randomly chosen patients were evaluated blindly by a second radiologist with specialisation in liver imaging >6 years.

### Liver function analysis

Baseline albumin, bilirubin, international normalised ratio (INR) and albumin–bilirubin (ALBI) score were recorded for each patient. Liver decompensation was defined as a grade 2 bilirubin increase according to CTCAE (Common Terminology Criteria for Adverse Events) version 5.0, and time to liver decompensation was recorded. In summary, grade 2 bilirubin increase was defined as bilirubin >30 µmol/l if the baseline value was normal or more than 1.5-fold increase if the baseline was abnormal. Patients without any follow-up bilirubin were excluded from this analysis. Patients with no grade 2 bilirubin increase were censored at the last available follow-up.

### Statistical analysis

For all image-based criteria and additional clinical variables, univariate Cox proportional hazard regression was performed using R statistical software (R version 3.6.3). Categorical variables were used as is; continuous variables were scaled to mean value zero and standard deviation one. For multivariate analysis, variable selection based on the Least Absolute Shrinkage and Selection Operator (LASSO) was performed using the R package glmnet version 4.0 [18]. The regularisation parameter was chosen as the value that minimised the mean cross-validated error in 10-fold cross-validation. The LASSO allows the selection of variables by shrinking down to zero coefficient weights for variables nonrelated to the outcome. Then, parameters with nonzero coefficient weights were integrated into a multivariable logistic regression analysis. Associated hazard ratios and 95% confidence intervals were estimated. Due to the large number of missing cases, parameters and criteria derived from diffusion-weighted MRI were excluded from multivariate analysis. These analyses were performed independently for the endpoints of OS and liver decompensation. Interreader variation of imaging features was calculated by using Cohen simple κ-statistic with 95% confidence intervals and was interpreted as follows: 0–0.20, slight agreement; 0.21–0.40, fair agreement; 0.41–0.60, moderate agreement; 0.61– 0.80, substantial agreement; and 0.81– 1.00, almost perfect agreement.

## Results

### Patient and baseline imaging characteristics

A total of 376 patients [median age, 66 years; interquartile range (IQR), 60–73; 326 men] were included in this post hoc analysis. Baseline characteristics of the patients are summarised in Table 1. While 192 (51.1%) patients were randomised to combination therapy, 184 (48.9%) were randomised to sorafenib monotherapy. Three hundred and forty-three (91.2%) patients had Child–Pugh A liver function and 295 (78.4%) patients had cirrhosis. The most common aetiology was alcoholic liver disease in 216 (57.4%) patients, followed by hepatitis C in 91 (24.2%) and hepatitis B in 36 (9.5%) patients. BCLC stage was A in 8 (2.1%), B in 112 (29.8%) and C in 256 (68.1%) patients. The mean baseline albumin and total bilirubin values of the study population were 37.6 ± 8.1 and 15.5 ± 7.6 mg/dl, and the mean ALBI score was −2.45 ± 0.73. At the end of the study, 312 patients were deceased, and the median OS of the cohort was 12.1 months.

**Table 1:** Baseline characteristics and imaging features of patients.

Variable	
Randomisation	
Sorafenib	184 (48.9%)
SIRT + sorafenib	192 (51.1%)
Sex	
Female	50 (13.2%)
Male	326 (86.7%)
Age	
Mean (SD)	66.0 (8.5)
Median (IQR)	66.0 (13)
Liver cirrhosis	
Missing	6 (1.5%)
No	75 (19.9%)
Yes	295 (78.4%)
Alcohol	
No	160 (42.5%)
Yes	216 (57.4%)
Max. diameter of the largest lesion (mm)	
CT, mean (SD)	70.5 (40.7)
MRI, mean (SD)	71.8 (41.3)
Macrovascular invasion	
CT—yes	192 (51.0%)
MR—yes	183 (50.8%)
Presence of atypical HCC lesion	
CT—yes	193 (51.3%)
MR—yes	181 (50.2%)
Biliary dilatation	
CT—yes	25 (6.6%)
MR—yes	24 (6.5%)
Varices	
CT—yes	100 (26.5%)
MR—yes	97 (26.6%)
Ascites	
CT—yes	61 (16.2%)
MR—yes	59 (16.2%)
Pleural effusion	
CT—yes	9 (2.3%)
MR—yes	9 (2.4%)
Complete capsule	
CT—yes	177 (47.2%)
MR—yes	174 (48.4%)
Intratumoral fat deposition	
CT—yes	52 (14.0%)
MR—yes	51 (14.6 %)
Satellite lesion	
CT—yes	151 (40.1%)
MR—yes	139 (38.1%)
Peritumoral arterial enhancement	
CT—yes	181 (48.2%)
MR—yes	139 (40.4%)
Peritumoral hypointensity in the hepatobiliary phase	
Yes	163 (46.1%)
Signal intensity of tumour in hepatobiliary phase, low	299 (84.4%)
Signal intensity of tumour on DWI, high	199 (95.6%)
Liver–spleen ratio (LSR), >1.5	219 (87.6%)
HU of tumour on native images, mean (SD)	41.5 (15.7)
HU of tumour on arterial phase, mean (SD)	73.6 (24.0)
HU of tumour on portal phase, mean (SD)	88.4 (21.5)
HU of tumour on venous phase, mean (SD)	80.2 (18.4)
ADC value of tumour, mean (SD)	924.6 (252.9)
Relative liver enhancement (RLE), mean (SD)	1.01 (1.1)
Baseline albumin (g/dl), mean (SD)	37.6 (8.1)
Baseline bilirubin (mg/dl), mean (SD)	15.5 (7.6)
Baseline ALBI score, mean (SD)	−2.45 (0.73)
Baseline INR, mean (SD)	1.14 (0.3)

Max. diameter of the largest lesion (mm).

*ADC* apparent diffusion coefficient, *ALBI* albumin–bilirubin, *DWI* diffusion-weighted imaging, *HCC* hepatocellular carcinoma, *HU* Hounsfield unit, *INR* International normalised ratio, *IQR* interquartile range, *SD* standard deviation.

The median interval between baseline CT imaging and randomisation was 7 (IQR, 2–15) days. The median time interval between baseline CT and MRI was 1 (IQR, 0–13) day. The median diameter of the largest measurable lesion on CT was 63 mm (IQR 38–93 mm) and on MRI was 71.8 mm (IQR 39–95 mm). The detection rates of imaging features by CT and MRI were 51.0% and 50.8% for macrovascular invasion; 51.3% and 50.2% for atypical HCC lesions; 6.6 and 6.5% for biliary dilatation; 26.5 and 26.6% for varices; 16.2 and 16.2% for ascites; 2.3 and 2.4% for pleural effusion; 47.2 and 48.4% for complete capsule; 14.0 and 14.6% for intratumoral fat deposition; 40.1 and 38.1% for satellite lesions; and 48.2 and 40.4% for peritumoral arterial enhancement (exemplary images for several parameters are given in Supplementary Fig. 2). Overall, CT and MRI showed moderate to excellent concordance to detect the same imaging feature (*κ* values: 0.77–1). Considering MRI-specific parameters in the hepatobiliary phase, 46.1% of the patients had peritumoral hypointensity and the lesion was hypointense in 84.4%. LSR was >1.5 in 87.6% of the patients.

The interreader agreement for the individual imaging features in CT ranged from moderate to almost perfect (*κ* = 0.49–0.88), and also in MRI ranged from moderate to almost perfect (*κ* = 0.50–0.96). For most imaging features (15/17 in CT and 13/17 in MRI), interreader agreement was substantial or almost perfect (Supplementary Table 1).

### Association between imaging or clinical features and OS

**Fig. 1:**
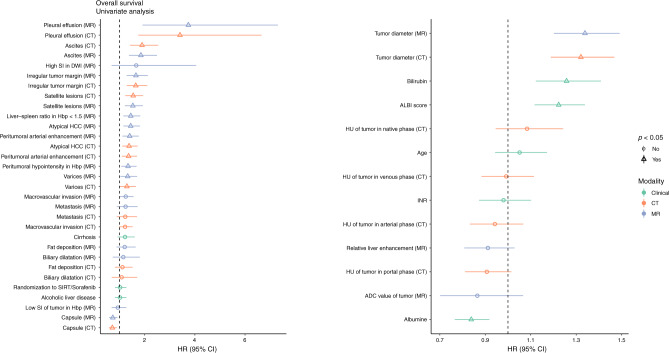
Effect of Various Clinical and Imaging Features on Overall Survival. The left side shows the categorical and the right side the continuous variables. Statistical significance is represented with a triangle. The green color shows clinical, orange CT-based, and purple MR-based parameters.

The prognostic value of the baseline radiological and clinical parameters in terms of OS is displayed in Fig. 1 and Supplementary Table 2. In univariate analysis, out of the parameters related to tumour biology, the presence of capsule was associated with better survival, while the presence of irregular margin, satellite lesions, atypical HCC lesions, peritumoral enhancement, peritumoral hypointensity in hepatobiliary phase, and larger tumour size on both imaging modalities were associated with lower OS. Out of the imaging parameters related to liver function, pleural effusion, ascites, varices and LSR < 1.5 were correlated with decreased OS. In addition, higher serum bilirubin, lower serum albumin and increased ALBI score from the clinical parameters were associated with worse outcomes.

There was no significant correlation between the OS and the treatment arm (sorafenib monotherapy vs combination of SIRT and sorafenib). Further, the presence of vascular invasion, extrahepatic disease, biliary dilatation, or intratumoral fat deposition, tumour density expressed in HU units on different CT phases, age, INR value and presence of cirrhosis or alcoholic liver disease were not significant predictors for OS in both modalities. Also, in MRI-specific parameters, high SI in DWI images, low SI in hepatobiliary phase images and RLE did not have a correlation with OS.

**Fig. 2:**
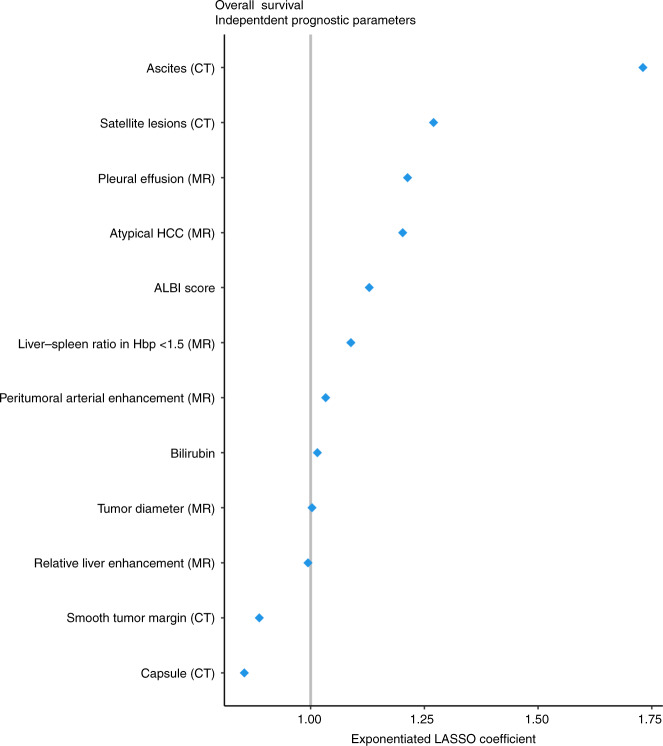
Multivariate Cox proportional hazards regression analysis of variables selected by LASSO for prediction of survival. Due to missing variables 298 patients were included in the multivariate analysis.

Multivariate analysis with LASSO-penalised logistic regression analysis identified 12 factors as independent predictors of OS (coefficient weights are listed in Supplementary Table 3). The presence of ascites, satellite lesions, pleural effusion, atypical HCC lesions, higher ALBI score, LSR < 1.5, presence of peritumoral arterial enhancement, higher bilirubin values and larger tumour size were associated with reduced OS, in order of decreasing hazard ratio; the presence of complete capsule, smooth margin and higher RLE were associated with better OS, also in order of decreasing hazard ratio (Fig. 2). We note that, while the ranking of predictors based on estimators is correct, the absolute values of estimators are biased and confidence intervals are not available; this is a well-known property of LASSO regression.

### Association between imaging features and liver decompensation

**Fig. 3:**
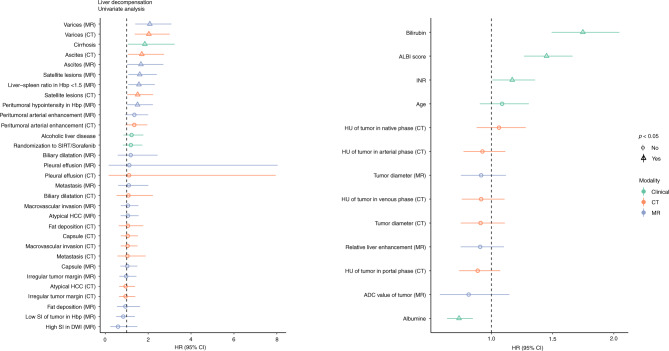
Effect of Various Clinical and Imaging Features on Liver Decompensation The left side shows the categorical and the right side the continuous variables. Statistical significance is represented with a triangle. The green color shows clinical, orange CT-based, and purple MR-based parameters.

For 300 of the patients included in the study, follow-up bilirubin levels were available and 113 patients had liver decompensation during the follow-up period. In univariate analysis, presence of varices, presence of cirrhosis, ascites, satellite lesions, LSR < 1.5 and peritumoral hypointensity in the hepatobiliary phase on the baseline images were associated with liver decompensation (Fig. 3 and Supplementary Table 4). Out of the clinical parameters, higher baseline bilirubin value, ALBI grade, INR value and lower albumin value were associated with liver decompensation.

**Fig. 4:**
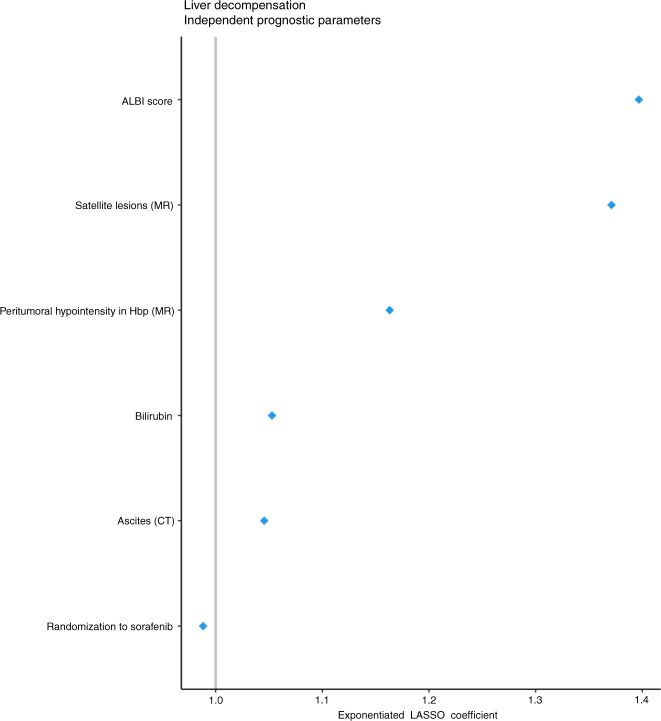
Multivariate Cox proportional hazards regression analysis of variables selected by LASSO for prediction of liver decompensation. Due to missing variables, 240 patients were included in the multivariate analysis.

LASSO-penalised logistic regression analysis identified six baseline factors as independent predictors of liver decompensation: higher ALBI score, presence of satellite lesions, presence of peritumoral hypointensity in the hepatobiliary phase, higher bilirubin values and ascites (in order of decreasing hazard ratio; coefficient weights are listed in Supplementary Table 5) were associated with liver decompensation; and randomisation to the sorafenib arm was associated with a lower rate of liver decompensation (Fig. 4).

## Discussion

This post hoc analysis of a randomised-controlled multicenter phase II trial represents the largest cohort to date to identify prognostic imaging biomarkers in patients with advanced HCC and provides the highest level of evidence published to date. Our results demonstrate that radiological imaging appearances, corresponding to worse liver function and aggressive tumour characteristics, determine OS after sorafenib treatment either alone or combined with SIRT. In this study, the presence of ascites, pleural effusion and LSR < 1.5 (determinants of compromised liver function) and the presence of satellite tumours, atypical HCC, relative liver enhancement, peritumoral arterial enhancement, larger tumour, irregular tumour margins and lack of complete capsule (determinants of invasive tumour behaviour or advanced stage of the tumour) were associated with poor outcome.

Sorafenib has been the primary treatment of advanced HCC cases since two studies showed a survival benefit of sorafenib therapy over placebo [3, 4]. Besides sorafenib, SIRT has been used in the treatment of advanced HCC cases. Although three randomised trials failed to show a survival benefit of SIRT (alone or combined with sorafenib) over sorafenib, a meta-analysis of these three studies demonstrated the non-inferiority of SIRT vs sorafenib [19]. However, patients classified as advanced HCC and allocated to sorafenib and/or SIRT do not benefit from these treatments equally and have a wide range of OS [7, 20]. Current classification systems (i.e. BCLC) do not consider imaging features, except size, number of lesions and presence of portal vein invasion.

Imaging features showing aggressive biological behaviour, such as the presence of peritumoral arterial enhancement [15], peritumoral hypointensity in hepatobiliary phase images [21], lack of complete capsule [22], irregular tumour margin [15] and eventually, presence of satellite nodules [11], imply spread of tumour cells beyond tumour borders and aggressive growth patterns. These features were correlated with poor OS in our study. These findings are in good agreement with the results of previous reports. However, these reports were from single-centre retrospective studies with relatively small sample sizes focusing on only some of these criteria [11, 15, 21]. The presence of satellite lesions and peritumoral hypointensity in the hepatobiliary phase has been shown to correlate with HCC recurrence after liver transplantation [11]. In a study evaluating survival of HCC patients who underwent TACE, irregular tumour margin and peritumoral enhancement on both CT and MRI were predictors of decreased survival besides vascular invasion [15].

mVI is an independent predictor of shorter recurrence-free survival after ablation, resection or transplantation [23–25]. The presence of peritumoral arterial enhancement, irregular tumour margin and peritumoral hypointensity in the hepatobiliary phase on preoperative MRI images has been shown to correlate with mVI in resected HCC samples [17, 21]. All of these three variables were associated with OS in our study.

Liver function is a key determinant of survival in HCC patients, and patients with preserved liver function benefit more from palliative treatments [9]. Besides laboratory function (albumin, bilirubin and ALBI score), imaging signs of decreased liver function, such as varices, ascites, pleural effusion and hepatocellular uptake of hepatobiliary contrast media (as described by LSR and RLE) were also predictors of OS in our study. LSR graded with a cut-off value of 1.5 has been shown to correlate with laboratory and clinical indicators of liver function, including ALBI or Child–Pugh grade [26, 27]. Baseline LSR with a cut-off value of 1.5 was a significant predictor of OS as well as of liver decompensation in our study. Besides LSR, baseline imaging features of peritumoral hypointensity in the hepatobiliary phase (descriptor of mVI), satellite lesions, ascites, and varices and baseline laboratory values of bilirubin, albumin, INR and ALBI score were correlated with deterioration in liver function during follow-up. As pointed out above, the presence of peritumoral hypointensity in the hepatobiliary phase and satellite lesions shows the aggressive biological behaviour of HCC and probably correlates with earlier progression and replacement of healthy liver tissue. Liver decompensation was determined as a grade 2 bilirubin increase in our analysis because it was an exclusion criterion of the study and also is a sign of radiation-induced liver damage [28]. In LASSO analysis, there was a trend to less liver decompensation for patients in the sorafenib arm. This analysis was made on the intention-to-treat (ITT) population, rather than the per-protocol (PP) population, in order not to lose too many patients. However, a separate analysis done only on the PP population identified similar results (data not shown).

Another result of our study was that CT and MRI had similar prognostic values using the variables that are evaluable on both. MRI has superior contrast resolution than CT, and the diagnostic arm of the SORAMIC trial has shown that MRI with hepatocyte-specific contrast agents provides more accurate treatment decisions than CT [14]. Despite the different detection rates of some radiological parameters between CT and MRI in our study, the difference was slight and did not translate to a difference in endpoints, such as OS and liver decompensation. However, MRI allows the detection of additional parameters, which were significantly correlated with both OS and liver decompensation in our study (LSR, mVI). Thus, MRI most likely has a higher value in prognostic prediction of HCC patients.

A notable finding in our study was that both ADC values and DWI SI of the lesion were not correlated with OS in univariate analysis. In SORAMIC, DWI was not mandatory for screening and not standardised regarding *b* values, and for almost half of the patients included in this analysis, DWI images were not available. This situation might be the reason for the missing correlation. Nevertheless, despite some studies reporting a correlation between lower ADC values with early recurrence after liver transplantation [10] or SIRT [29], and lower OS after TACE or SIRT [30], all of these studies are retrospective single-centre studies; and other studies have reported no such correlation [15]. HCC has mostly a heterogeneous intratumoral architecture, especially in large lesions. This heterogeneity may be responsible for these discrepant results. In addition, in our study, images were obtained with various brand scanners using different *b* values, ADC values were measured in a single slice, not volumetrically and only mean ADC values were recorded.

Besides DWI-related factors, the presence of fat deposition inside the tumour, biliary dilatation, macrovascular invasion, extrahepatic tumour and tumours with low SI in the hepatobiliary phase at baseline were not significant prognostic factors in our analysis. Lack of correlation between OS and macrovascular invasion or extrahepatic metastasis underlines the importance of new imaging biomarkers. A post hoc analysis of the SORAMIC trial has also shown that the presence of extrahepatic disease at baseline does not influence survival in liver-dominant HCC patients (without lung involvement) [31]. Some studies have reported the prognostic importance of fat deposition [32], biliary invasion [33] and low SI of lesions in the hepatobiliary phase [34]; however, most of these studies are in patients with early HCC and there are conflicting results in the literature, especially in patients with intermediate or advanced HCC [15]. Our results may imply that these factors lack prognostic differentiation ability in advanced stages.

This study has some limitations. First, except for the presence of atypical lesions, the imaging features of only the index lesion were evaluated. Second, there were missing series in some patients, and DWI was not available in almost half of the patients; thus, the diffusion-based features had to be excluded from the multivariate analysis. Also, this was a post hoc analysis and MRI findings need to be confirmed in patients who received extracellular contrast agents. However, our study presents large-scale imaging data from a prospective trial obtained with different scanner brands, but with a defined image protocol and concise clinical follow-up assessment [14].

Our study provides an appropriate basis for future clinical researches. Future randomised-controlled trials should use imaging features for patient stratification. Further on, after validation, these imaging features should be taken into consideration in the development of new classification systems. Recently, atezolizumab–bevacizumab combination has been approved in the first-line treatment of HCC with significantly improved OS and progression-free survival compared to sorafenib in the Imbrave trial [35]. However, this combination therapy has shown to be not cost-effective as sorafenib [36]. These imaging features could serve in patient selection for more aggressive and effective therapies after validating in cohorts with other systemic therapies. In addition, retrospective studies comparing the effects of treatment methods on HCC should match groups according to imaging features influencing survival. In addition, some new markers correlated with outcome in HCC patients have been described recently (i.e. circulating free DNA) [37], and further evaluation of possible correlations between such markers and imaging features should be warranted.

In conclusion, beyond established features, a number of radiological imaging features related to liver function as well as tumour extent and aggressiveness at baseline can predict survival and liver decompensation after sorafenib and SIRT in patients with HCC. These imaging features have similar prognostic value on both CT and MRI.

### Reporting summary

Further information on experimental design is available in the Nature Research Reporting Summary linked to this paper.

## Supplementary information

Supplementary figure 1

Supplementary figure 2

Supplementary tables

Reporting summary

## Data Availability

The datasets generated and analysed during the current study are available from the corresponding author on reasonable request.
