# Supplementary figures and images for: Prognostic value of baseline imaging and clinical features in patients with advanced hepatocellular carcinoma
Source: Br J Cancer. 2021 Oct 22;126(2):211–8. doi: 10.1038/s41416-021-01577-6 (PMC8770679; doi:10.1038/s41416-021-01577-6)

## Supplementary figure 1. CONSORT diagram

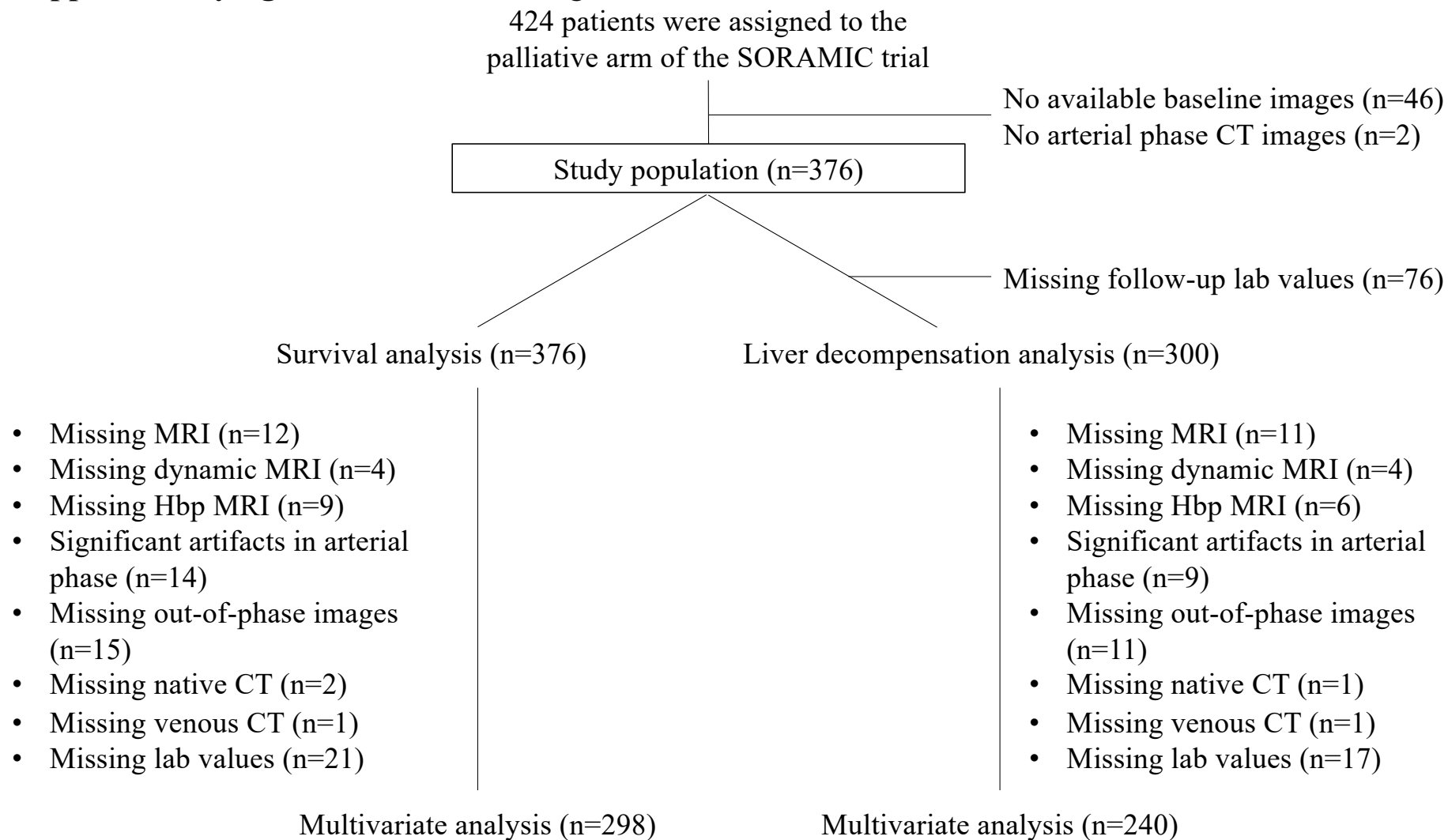

Supplement: Supplementary file 1 — Supplementary figure 1 [file 41416_2021_1577_MOESM1_ESM.pdf]
