# Supplementary material for: Prognostic value of baseline imaging and clinical features in patients with advanced hepatocellular carcinoma
Source: Br J Cancer. 2021 Oct 22;126(2):211–8. doi: 10.1038/s41416-021-01577-6 (PMC8770679; doi:10.1038/s41416-021-01577-6)

Supplementary figure 2. Examples for imaging parameters

A. Irregular tumor margin

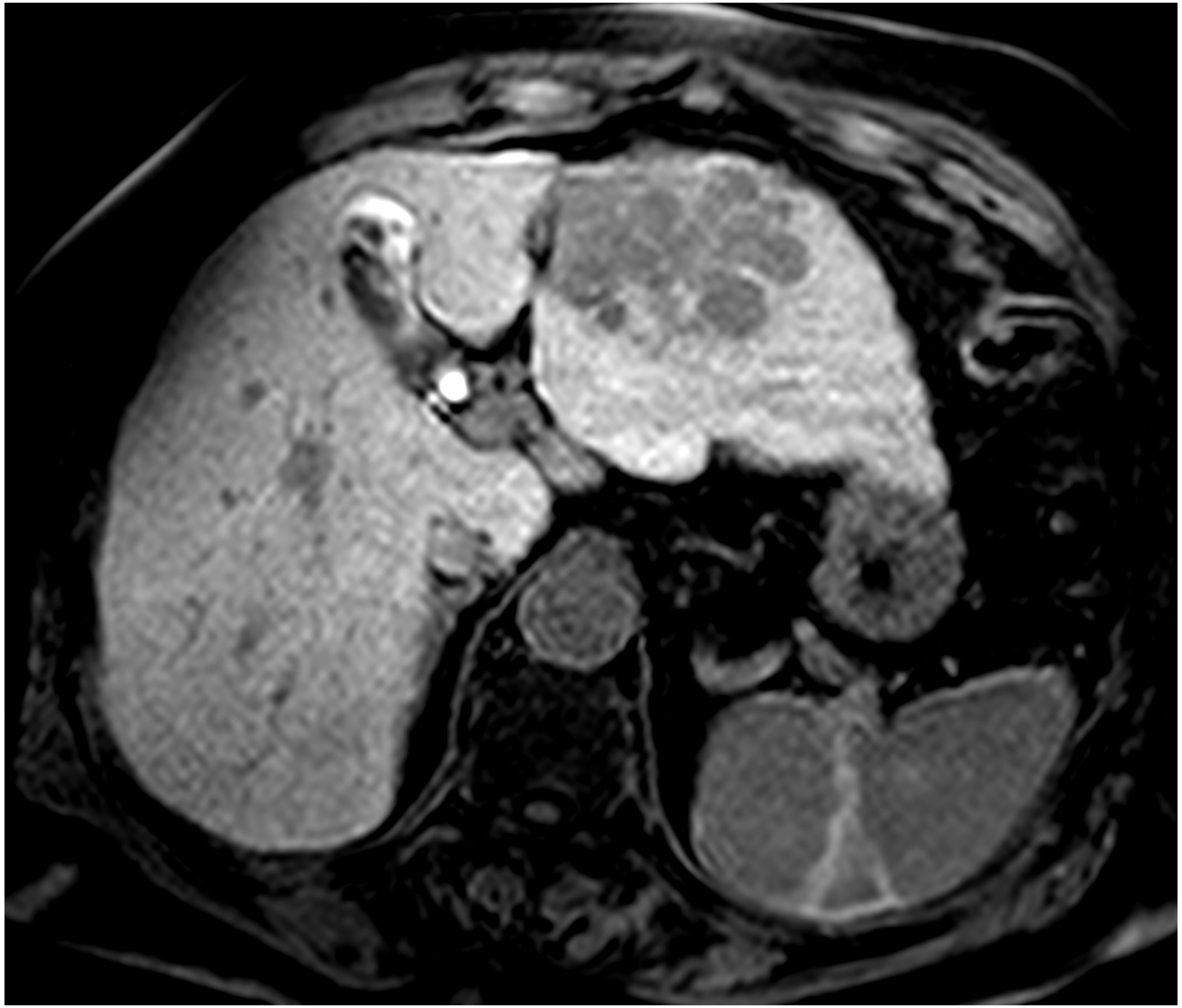

B. Peritumoral arterial enhancement

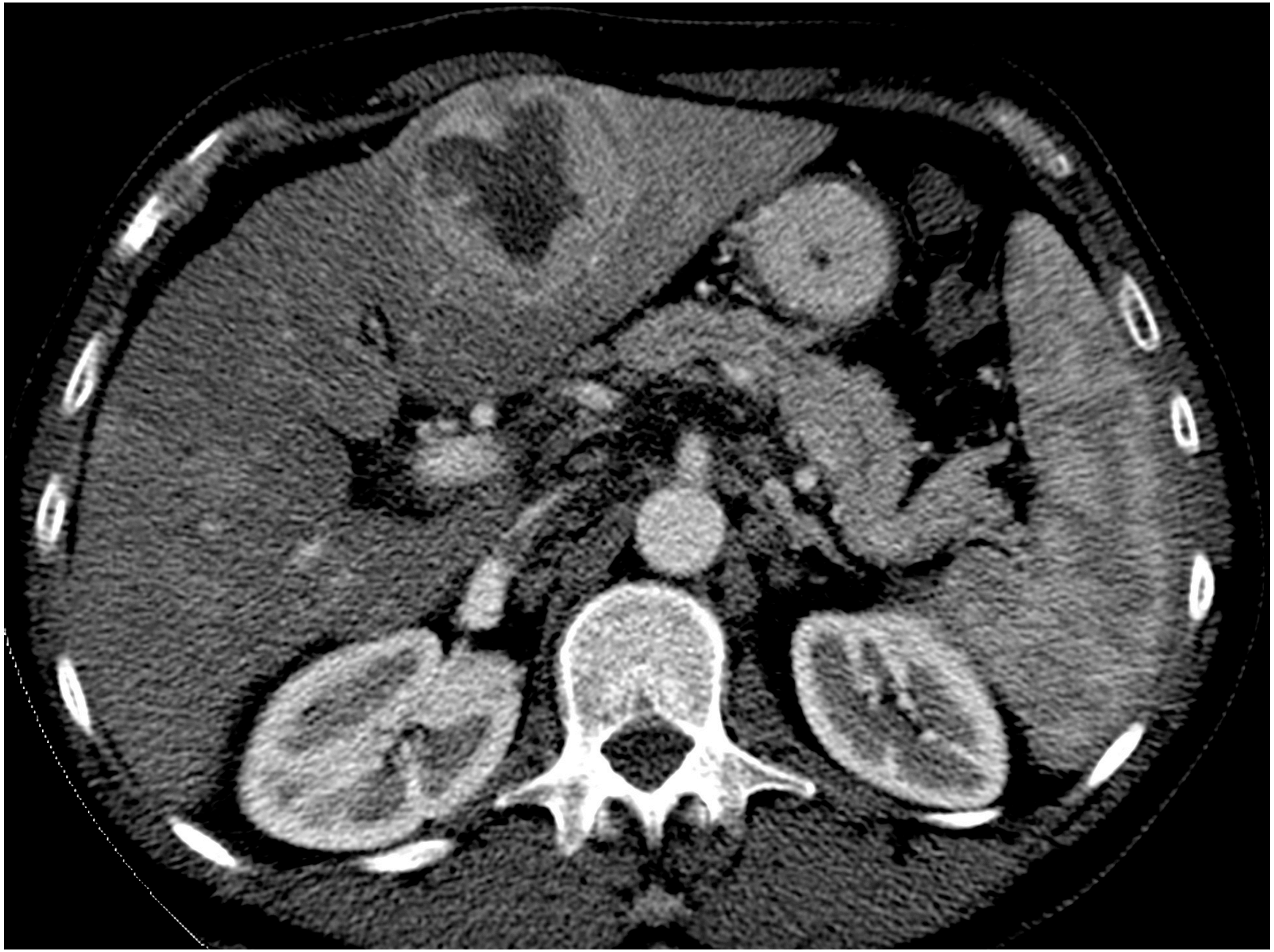

C. Satellite lesions

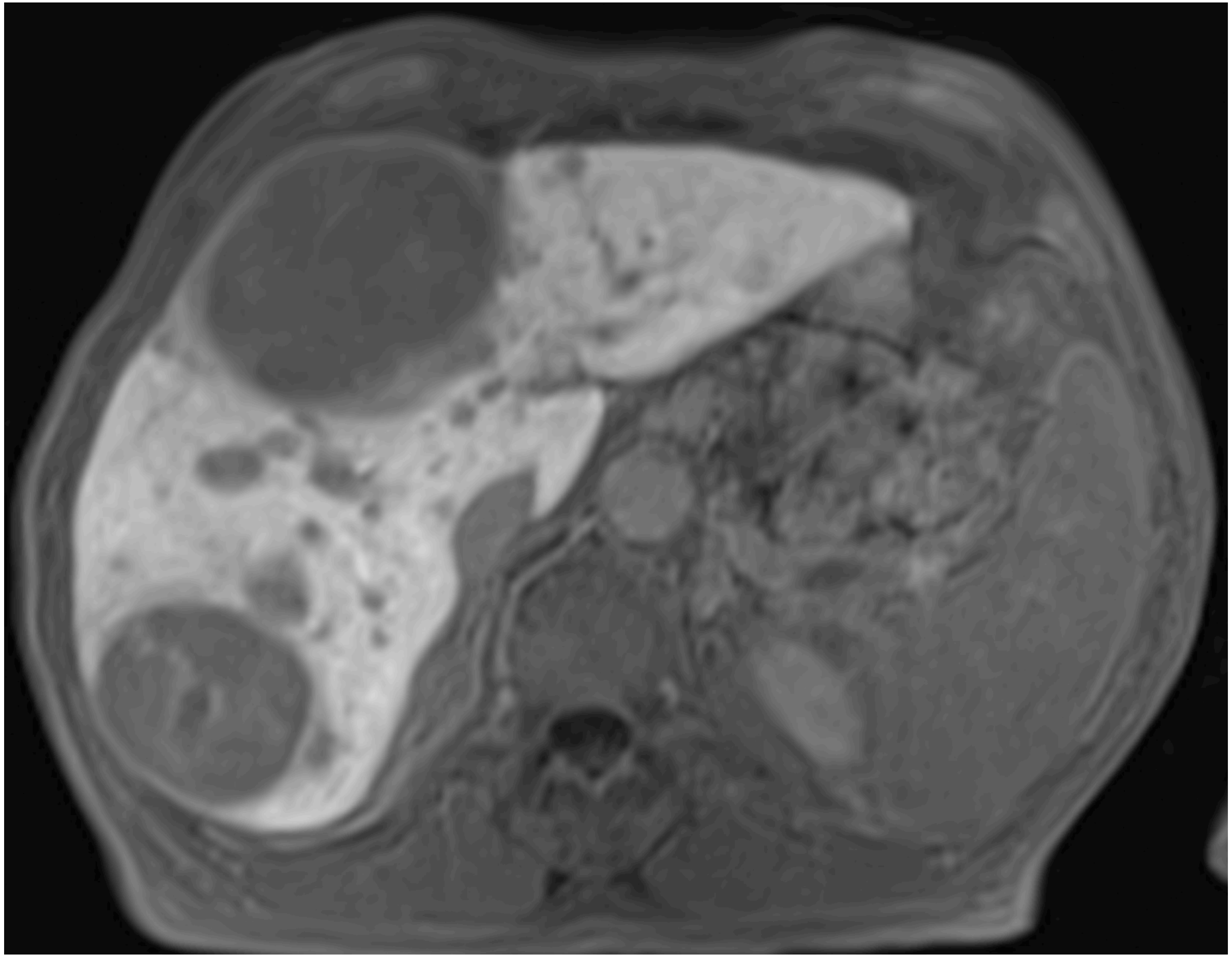

D. Peritumoral hypointensity in hepatobiliary phase

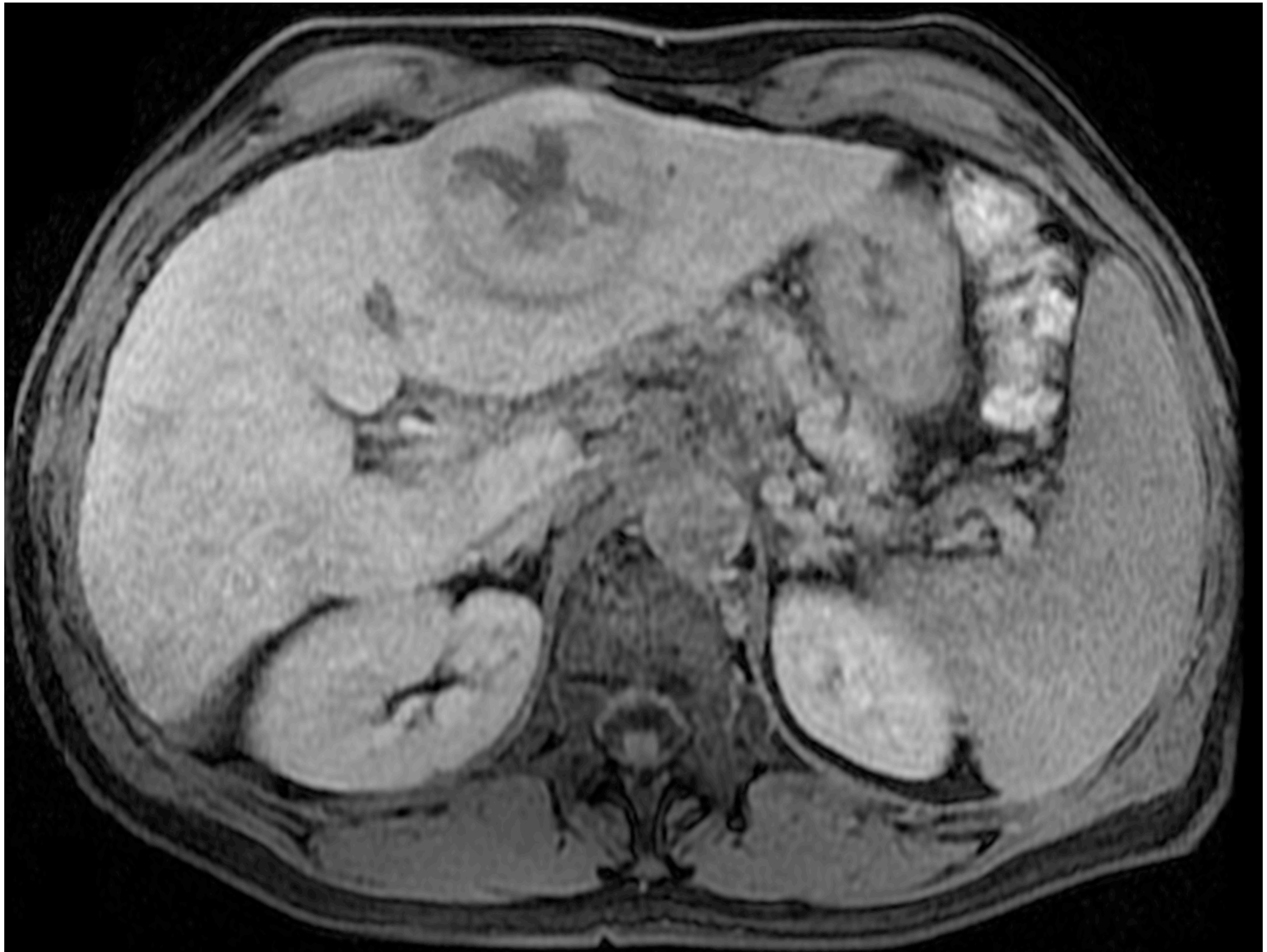

E. Intratumoral fat

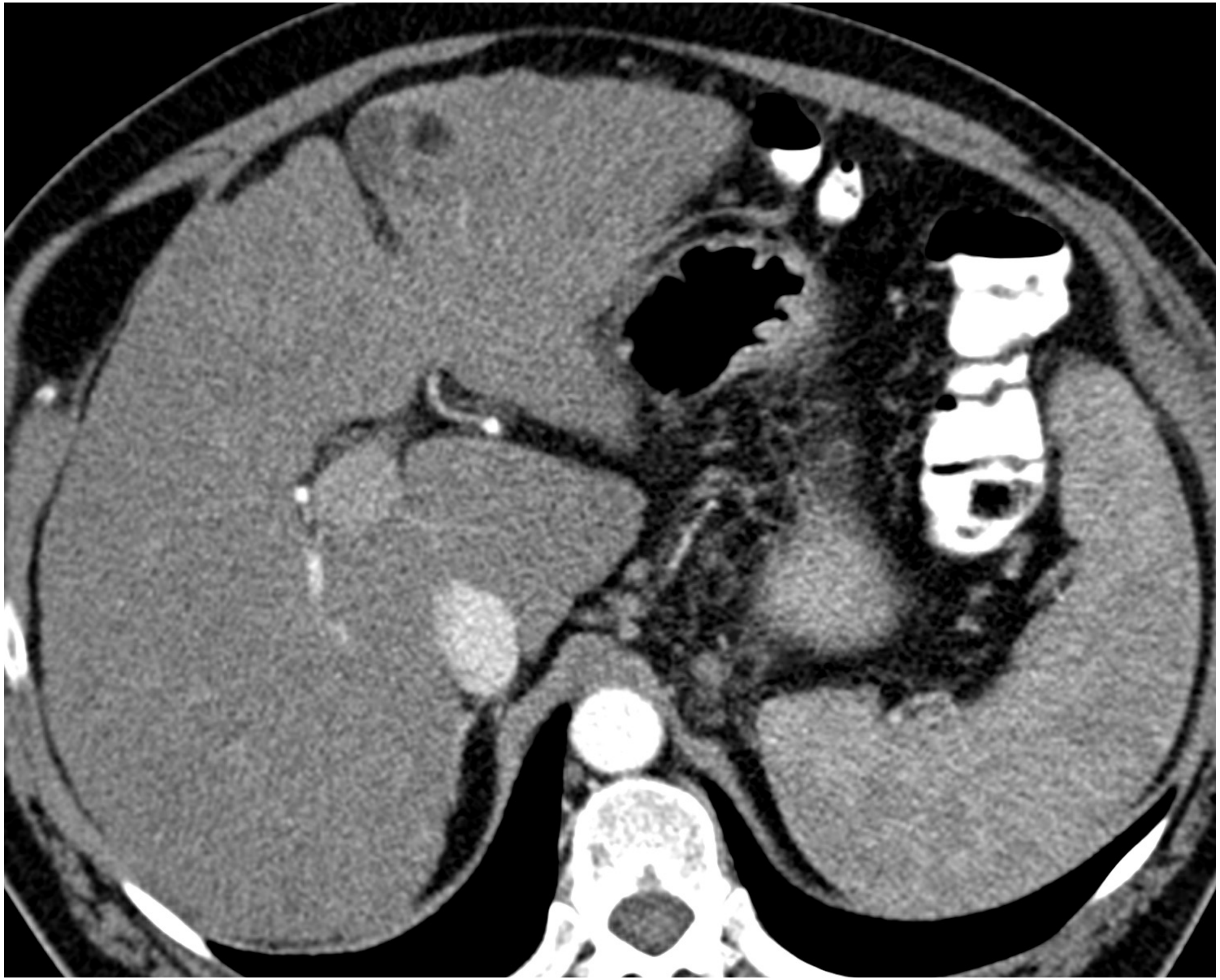

F. Capsule

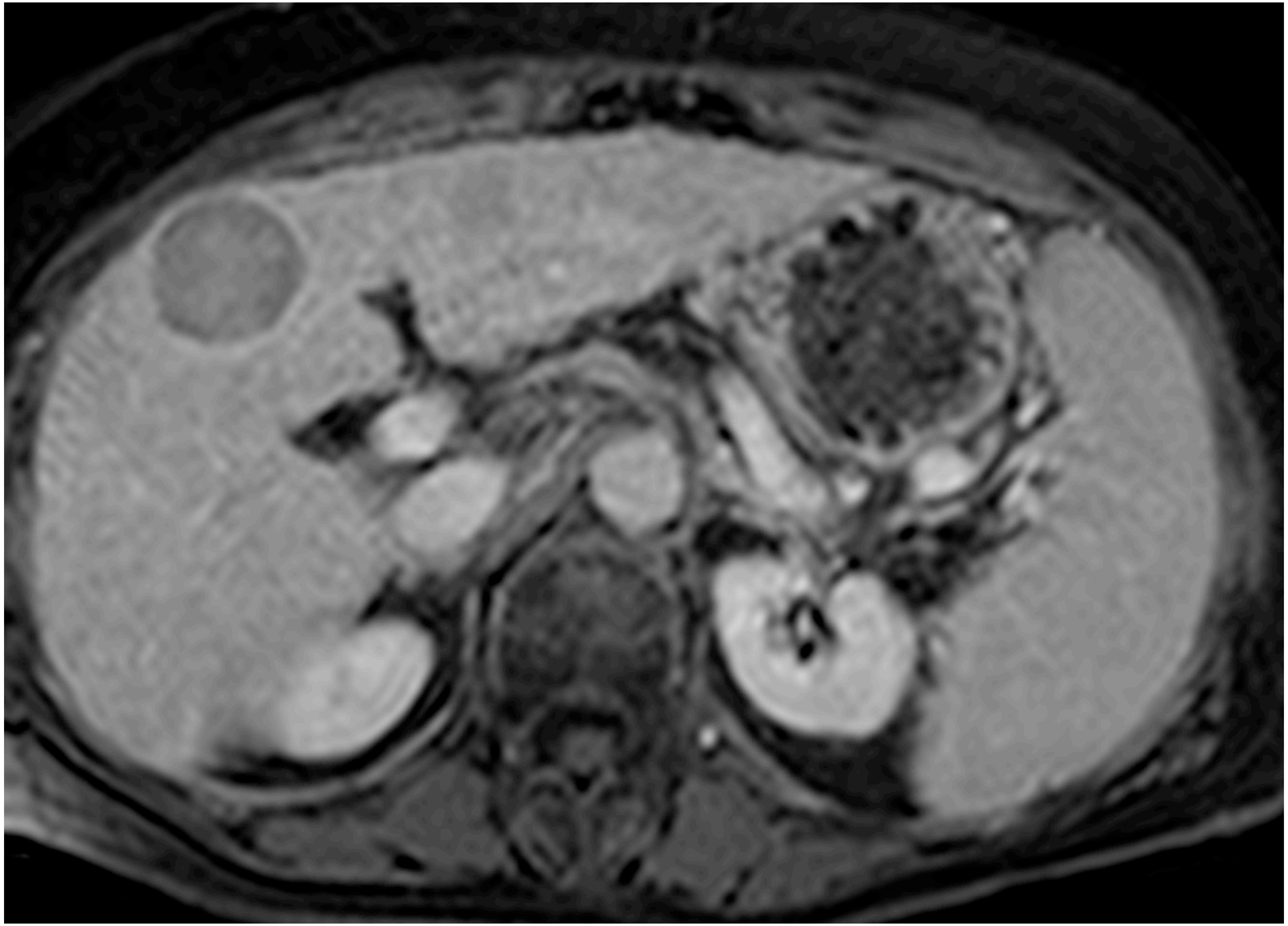

G. Atypical HCC

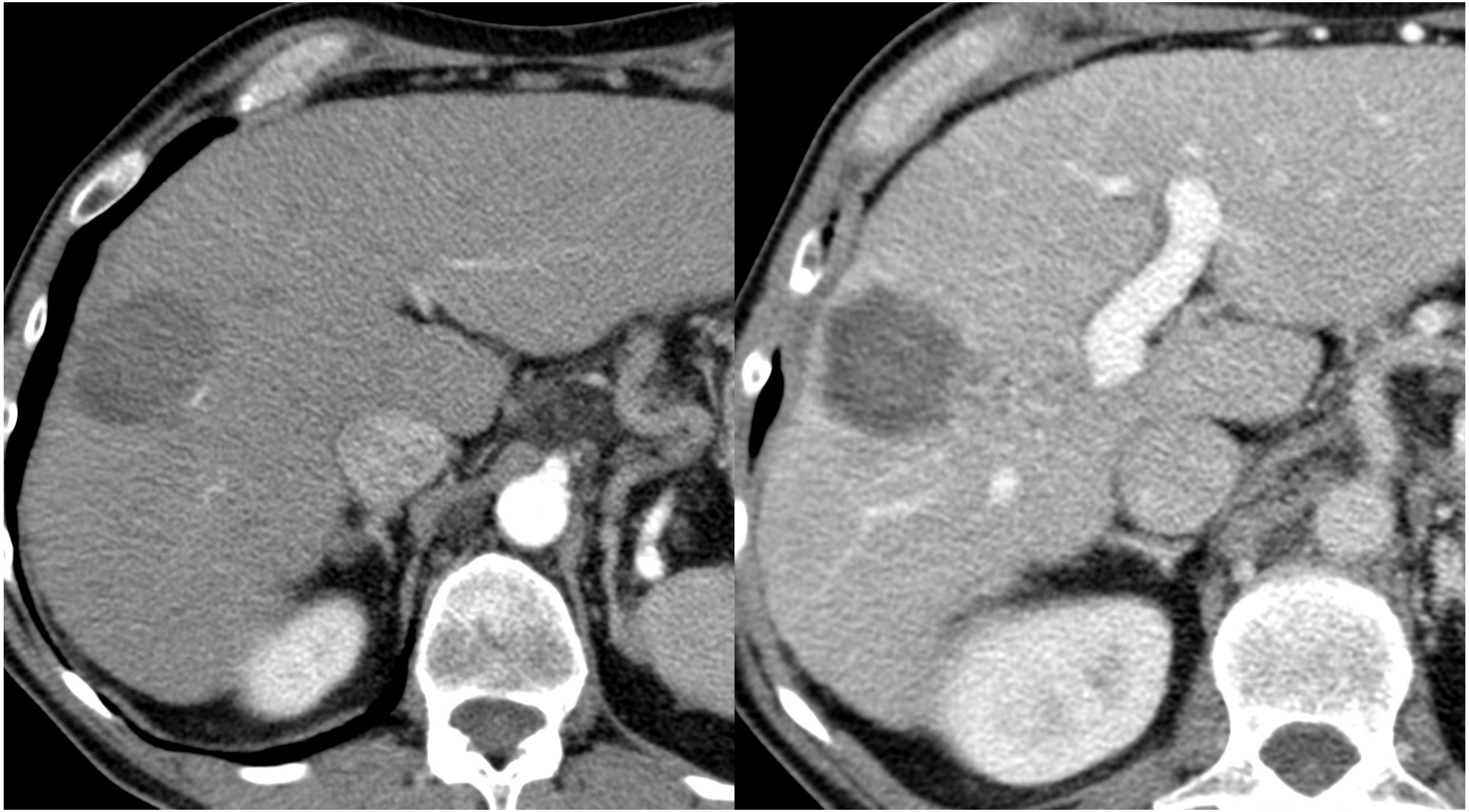

Supplement: Supplementary file 2 — Supplementary figure 2 [file 41416_2021_1577_MOESM2_ESM.pdf]
