# Supplementary material for: Prognostic value of baseline imaging and clinical features in patients with advanced hepatocellular carcinoma
Source: Br J Cancer. 2021 Oct 22;126(2):211–8. doi: 10.1038/s41416-021-01577-6 (PMC8770679; doi:10.1038/s41416-021-01577-6)
Supplement: Supplementary file 3 — Supplementary tables [file 41416_2021_1577_MOESM3_ESM.pdf]

**Supplementary table 1. Interreader agreement**

| **Imaging features** | **κ Value** |
|---|---|
| Tumor diameter (CT) | 0.84 |
| Atypical HCC (CT) | 0.70 |
| Macrovascular invasion (CT) | 0.88 |
| Biliary dilatation (CT) | 0.75 |
| Metastasis (CT) | 0.59 |
| Varices (CT) | 0.73 |
| Ascites (CT) | 0.81 |
| Pleural effusion (CT) | 0.82 |
| Capsule (CT) | 0.68 |
| Intratumoral fat (CT) | 0.49 |
| Irregular tumor margin (CT) | 0.75 |
| Satellite lesions (CT) | 0.67 |
| Peritumoral arterial enhancement (CT) | 0.7 |
| HU of tumor in native phase | 0.75 |
| HU of tumor in arterial phase | 0.80 |
| HU of tumor in portal phase | 0.64 |
| HU of tumor in venous phase | 0.70 |
| Tumor diameter (MR) | 0.84 |
| Atypical HCC (MR) | 0.68 |
| Macrovascular invasion (MR) | 0.88 |
| Biliary dilatation (MR) | 0.75 |
| Metastasis (MR) | 0.59 |
| Varices (MR) | 0.65 |
| Ascites (MR) | 0.77 |
| Pleural effusion (MR) | 0.69 |
| Capsule (MR) | 0.64 |
| Intratumoral fat (MR) | 0.6 |
| Irregular tumor margin (MR) | 0.79 |
| Satellite lesions (MR) | 0.66 |
| Peritumoral arterial enhancement (MR) | 0.68 |
| Peritumoral hypointensity in hepatobiliary phase (MR) | 0.5 |
| Signal intensity of tumor in hepatobiliary phase (low vs. others) (MR) | 0.6 |
| Signal intensity of tumor in precontrast images (MR) | 0.96 |
| Signal intensity of tumor in hepatobiliary phase (MR) | 0.88 |

**Supplementary table 2. Univariate analysis of correlation between multiple variables and overall survival**

| Parameter | HR | p-value | CI low | CI high |
|---|---|---|---|---|
| **Pleural effusion (MR)** | **3.74** | **0.000** | **1.91** | **7.31** |
| **Pleural effusion (CT)** | **3.41** | **0.000** | **1.74** | **6.66** |
| **Ascites (CT)** | **1.89** | **0.000** | **1.41** | **2.54** |
| **Ascites (MR)** | **1.85** | **0.000** | **1.37** | **2.49** |
| High SI in DWI (MR) | 1.66 | 0.265 | 0.68 | 4.05 |
| **Irregular tumor margin (MR)** | **1.65** | **0.000** | **1.28** | **2.12** |
| **Irregular tumor margin (CT)** | **1.64** | **0.000** | **1.28** | **2.10** |
| **Satellite lesions (CT)** | **1.54** | **0.000** | **1.23** | **1.94** |
| **Satellite lesions (MR)** | **1.53** | **0.000** | **1.21** | **1.93** |
| **Liver-spleen ratio in Hbp <1.5 (MR)** | **1.44** | **0.002** | **1.14** | **1.82** |
| **Atypical HCC (MR)** | **1.44** | **0.002** | **1.15** | **1.81** |
| **Peritumoral arterial enhancement (MR)** | **1.40** | **0.004** | **1.11** | **1.77** |
| **Atypical HCC (CT)** | **1.38** | **0.005** | **1.10** | **1.72** |
| **Peritumoral arterial enhancement (CT)** | **1.36** | **0.007** | **1.09** | **1.69** |
| **Tumor diameter (MR)** | **1.34** | **0.000** | **1.20** | **1.49** |
| **Peritumoral hypointensity in Hbp (MR)** | **1.33** | **0.013** | **1.06** | **1.68** |
| **Varices (MR)** | **1.32** | **0.029** | **1.03** | **1.69** |
| **Tumor diameter (CT)** | **1.32** | **0.000** | **1.19** | **1.47** |
| **Varices (CT)** | **1.29** | **0.044** | **1.01** | **1.65** |
| **Bilirubin** | **1.26** | **0.000** | **1.12** | **1.41** |
| Macrovascular invasion (MR) | 1.24 | 0.059 | 0.99 | 1.56 |
| Metastasis (MR) | 1.24 | 0.203 | 0.89 | 1.72 |
| Metastasis (CT) | 1.22 | 0.221 | 0.89 | 1.69 |
| **ALBI score** | **1.22** | **0.000** | **1.12** | **1.34** |
| Macrovascular invasion (CT) | 1.22 | 0.080 | 0.98 | 1.52 |
| Cirrhosis | 1.22 | 0.169 | 0.92 | 1.61 |
| Fat deposition (MR) | 1.20 | 0.251 | 0.88 | 1.64 |
| Biliary dilatation (MR) | 1.15 | 0.559 | 0.73 | 1.80 |
| Fat deposition (CT) | 1.11 | 0.499 | 0.82 | 1.52 |
| HU of tumor in native phase (CT) | 1.08 | 0.247 | 0.95 | 1.24 |
| Biliary dilatation (CT) | 1.08 | 0.740 | 0.69 | 1.70 |
| Age | 1.05 | 0.366 | 0.94 | 1.17 |
| Randomization to SIRT/sorafenib | 1.02 | 0.862 | 0.82 | 1.27 |
| Alcoholic liver disease | 1.02 | 0.885 | 0.81 | 1.27 |
| HU of tumor in venous phase (CT) | 0.99 | 0.894 | 0.88 | 1.11 |
| INR | 0.98 | 0.740 | 0.87 | 1.10 |
| HU of tumor in arterial phase (CT) | 0.94 | 0.352 | 0.83 | 1.07 |
| Low SI of tumor in Hbp (MR) | 0.93 | 0.647 | 0.68 | 1.27 |
| Relative liver enhancement (MR) | 0.91 | 0.135 | 0.81 | 1.03 |
| HU of tumor in portal phase (CT) | 0.91 | 0.089 | 0.81 | 1.01 |
| ADC value of tumor (MR) | 0.87 | 0.175 | 0.70 | 1.07 |
| **Albumin** | **0.84** | **0.000** | **0.77** | **0.92** |
| **Capsule (MR)** | **0.73** | **0.007** | **0.58** | **0.92** |
| **Capsule (CT)** | **0.71** | **0.003** | **0.57** | **0.89** |

**Bold type indicates statistical significance.**
ADC: Apparent diffusion coefficient, ALBI: Albumin-bilirubin, DWI: Diffusion weighted imaging, Hbp: Hepatobiliary phase, HCC: Hepatocellular carcinoma, HU: Hounsfield Unit, SI: Signal intensity, SIRT: Selective internal radiation therapy.

**Supplementary table 3. Coefficient weights of variables for overall survival analysis**

| Parameter | Coefficient | Exponentiated LASSO coefficient |
|---|---|---|
| Ascites (CT) | 0.548383169 | 1.730452905 |
| Satellite lesions (CT) | 0.238915913 | 1.269871753 |
| Pleural effusion (MR) | 0.193037968 | 1.212928845 |
| Atypical HCC (MR) | 0.184215502 | 1.202274888 |
| ALBI score | 0.121014154 | 1.128640887 |
| Liver-spleen ratio in Hbp >1.5 (MR) | 0.084637078 | 1.088322019 |
| Peritumoral arterial enhancement (MR) | 0.032552264 | 1.033087885 |
| Bilirubin | 0.01468812 | 1.014796521 |
| Tumor diameter (MR) | 0.00299551 | 1.003000001 |
| Tumor diameter (CT) | 0 | 1 |
| Atypical HCC (CT) | 0 | 1 |
| Macrovascular invasion (CT) | 0 | 1 |
| Biliary dilatation (CT) | 0 | 1 |
| Metastasis (CT) | 0 | 1 |
| Varices (CT) | 0 | 1 |
| Pleural effusion (CT) | 0 | 1 |
| Fat deposition (CT) | 0 | 1 |
| Peritumoral arterial enhancement (CT) | 0 | 1 |
| Macrovascular invasion (MR) | 0 | 1 |
| Biliary dilatation (MR) | 0 | 1 |
| Metastasis (MR) | 0 | 1 |
| Varices (MR) | 0 | 1 |
| Ascites (MR) | 0 | 1 |
| Capsule (MR) | 0 | 1 |
| Fat deposition (MR) | 0 | 1 |
| Smooth tumor margin (MR) | 0 | 1 |
| Satellite lesions (MR) | 0 | 1 |
| Peritumoral hypointensity in Hbp (MR) | 0 | 1 |
| Iso or high SI of tumor in Hbp (MR) | 0 | 1 |
| Age | 0 | 1 |
| Albumin | 0 | 1 |
| Cirrhosis | 0 | 1 |
| Alcoholic liver disease | 0 | 1 |
| Randomization to sorafenib | 0 | 1 |
| Relative liver enhancement (MR) | -0.005840161 | 0.994176859 |
| Smooth tumor margin (CT) | -0.119565001 | 0.88730633 |
| Capsule (CT) | -0.157447852 | 0.854321364 |

Please see Supplementary table 2 for the definition of abbreviations.

**Supplementary table 4. Univariate analysis of correlation between multiple variables and liver decompensation**

| Parameter | HR | p-value | CI low | CI high |
|---|---|---|---|---|
| **Varices (MR)** | **2.08** | **0.000** | **1.40** | **3.08** |
| **Varices (CT)** | **2.03** | **0.000** | **1.38** | **3.00** |
| **Cirrhosis** | **1.85** | **0.032** | **1.05** | **3.24** |
| **Bilirubin** | **1.75** | **0.000** | **1.49** | **2.05** |
| **Ascites (CT)** | **1.71** | **0.028** | **1.06** | **2.75** |
| **Ascites (MR)** | **1.67** | **0.040** | **1.02** | **2.71** |
| **Satellite lesions (MR)** | **1.61** | **0.020** | **1.08** | **2.40** |
| **Liver-spleen ratio in Hbp <1.5 (MR)** | **1.57** | **0.021** | **1.07** | **2.31** |
| **Satellite lesions (CT)** | **1.51** | **0.037** | **1.02** | **2.23** |
| **Peritumoral hypointensity in Hbp (MR)** | **1.50** | **0.040** | **1.02** | **2.22** |
| **ALBI score** | **1.45** | **0.000** | **1.27** | **1.66** |
| Peritumoral arterial enhancement (MR) | 1.36 | 0.122 | 0.92 | 2.00 |
| Peritumoral arterial enhancement (CT) | 1.36 | 0.109 | 0.93 | 1.97 |
| Alcoholic liver disease | 1.23 | 0.278 | 0.85 | 1.78 |
| Randomization to SIRT/Sorafenib | 1.19 | 0.358 | 0.82 | 1.73 |
| Biliary dilatation (MR) | 1.19 | 0.641 | 0.58 | 2.44 |
| **INR** | **1.17** | **0.038** | **1.01** | **1.36** |
| Pleural effusion (MR) | 1.12 | 0.910 | 0.16 | 8.04 |
| Pleural effusion (CT) | 1.11 | 0.919 | 0.15 | 7.95 |
| Metastasis (MR) | 1.10 | 0.761 | 0.60 | 2.01 |
| Age | 1.09 | 0.371 | 0.91 | 1.31 |
| Biliary dilatation (CT) | 1.08 | 0.834 | 0.53 | 2.22 |
| HU of tumor in native phase (CT) | 1.06 | 0.535 | 0.88 | 1.28 |
| Macrovascular invasion (MR) | 1.06 | 0.773 | 0.72 | 1.55 |
| Atypical HCC (MR) | 1.06 | 0.781 | 0.72 | 1.56 |
| Fat deposition (CT) | 1.05 | 0.841 | 0.63 | 1.77 |
| Capsule (CT) | 1.05 | 0.805 | 0.72 | 1.52 |
| Macrovascular invasion (CT) | 1.04 | 0.833 | 0.72 | 1.51 |
| Metastasis (CT) | 1.03 | 0.916 | 0.57 | 1.89 |
| Capsule (MR) | 1.02 | 0.902 | 0.70 | 1.50 |
| Irregular tumor margin (MR) | 0.98 | 0.910 | 0.66 | 1.46 |
| Atypical HCC (CT) | 0.95 | 0.787 | 0.65 | 1.38 |
| Irregular tumor margin (CT) | 0.95 | 0.787 | 0.64 | 1.40 |
| Fat deposition (MR) | 0.94 | 0.822 | 0.54 | 1.63 |
| HU of tumor in arterial phase (CT) | 0.93 | 0.418 | 0.77 | 1.11 |
| MR_maxSize_tm | 0.92 | 0.390 | 0.75 | 1.12 |
| HU of tumor in venous phase (CT) | 0.92 | 0.366 | 0.76 | 1.11 |
| Tumor diameter (CT) | 0.91 | 0.358 | 0.75 | 1.11 |
| Relative liver enhancement (MR) | 0.91 | 0.331 | 0.75 | 1.10 |
| HU of tumor in portal phase (CT) | 0.89 | 0.217 | 0.74 | 1.07 |
| Low SI of tumor in Hbp (MR) | 0.84 | 0.492 | 0.51 | 1.38 |
| ADC value of tumor (MR) | 0.81 | 0.239 | 0.58 | 1.15 |
| **Albumin** | **0.74** | **0.000** | **0.64** | **0.85** |
| High SI in DWI (MR) | 0.59 | 0.269 | 0.24 | 1.49 |

**Bold type indicates statistical significance.**
Please see Supplementary table 2 for the definition of abbreviations.

**Supplementary table 5. Coefficient weights of variables for liver decompensation analysis**

| Parameter | Coefficient | Exponentiated LASSO coefficient |
|---|---|---|
| ALBI score | 0.334221647 | 1.396852718 |
| Satellite lesions (MR) | 0.315626427 | 1.371117948 |
| Peritumoral hypointensity in Hbp (MR) | 0.151116445 | 1.163132091 |
| Bilirubin | 0.051506011 | 1.052855515 |
| Ascites (CT) | 0.04456833 | 1.045576419 |
| Tumor diameter (CT) | 0 | 1 |
| Atypical HCC (CT) | 0 | 1 |
| Macrovascular invasion (CT) | 0 | 1 |
| Biliary dilatation (CT) | 0 | 1 |
| Metastasis (CT) | 0 | 1 |
| Varices (CT) | 0 | 1 |
| Pleural effusion (CT) | 0 | 1 |
| Capsule (CT) | 0 | 1 |
| Fat deposition (CT) | 0 | 1 |
| Smooth tumor margin (CT) | 0 | 1 |
| Satellite lesions (CT) | 0 | 1 |
| Peritumoral arterial enhancement (CT) | 0 | 1 |
| Tumor diameter (MR) | 0 | 1 |
| Atypical HCC (MR) | 0 | 1 |
| Macrovascular invasion (MR) | 0 | 1 |
| Biliary dilatation (MR) | 0 | 1 |
| Metastasis (MR) | 0 | 1 |
| Varices (MR) | 0 | 1 |
| Ascites (MR) | 0 | 1 |
| Pleural effusion (MR) | 0 | 1 |
| Capsule (MR) | 0 | 1 |
| Fat deposition (MR) | 0 | 1 |
| Smooth tumor margin (MR) | 0 | 1 |
| Peritumoral arterial enhancement (MR) | 0 | 1 |
| Iso or high SI of tumor in Hbp (MR) | 0 | 1 |
| Liver-spleen ratio in Hbp <1.5 (MR) | 0 | 1 |
| Relative liver enhancement (MR) | 0 | 1 |
| Age | 0 | 1 |
| Albumin | 0 | 1 |
| Cirrhosis | 0 | 1 |
| Alcoholic liver disease | 0 | 1 |
| Randomization to sorafenib | -0.01177489 | 0.988294163 |

Please see Supplementary table 2 for the definition of abbreviations.
